# Plasmonic Spherical Nanoparticles Coupled with Titania Nanotube Arrays Prepared by Anodization as Substrates for Surface-Enhanced Raman Spectroscopy Applications: A Review

**DOI:** 10.3390/molecules26247443

**Published:** 2021-12-08

**Authors:** Jorge Jimenez-Cisneros, Juan Pablo Galindo-Lazo, Miguel Angel Mendez-Rojas, Jessica Rosaura Campos-Delgado, Monica Cerro-Lopez

**Affiliations:** Department of Chemical and Biological Sciences, University of the Américas Puebla, Sta. Catarina Mártir s/n, Cholula, Puebla 72810, Mexico; jorge.jimenezcs@udlap.mx (J.J.-C.); juan.galindolo@udlap.mx (J.P.G.-L.); miguela.mendez@udlap.mx (M.A.M.-R.); jessica.campos@udlap.mx (J.R.C.-D.)

**Keywords:** titania nanotubes, surface-enhanced Raman spectroscopy (SERS), silver nanoparticles, gold nanoparticles, platinum nanoparticles, plasmon resonance

## Abstract

As surface-enhanced Raman spectroscopy (SERS) continues developing to be a powerful analytical tool for several probes, four important aspects to make it more accessible have to be addressed: low-cost, reproducibility, high sensibility, and recyclability. Titanium dioxide nanotubes (TiO_2_ NTs) prepared by anodization have attracted interest in this field because they can be used as safe solid supports to deposit metal nanoparticles to build SERS substrate nanoplatforms that meet these four desired aspects. TiO_2_ NTs can be easily prepared and, by varying different synthesis parameters, their dimensions and specific features of their morphology can be tuned allowing them to support metal nanoparticles of different sizes that can achieve a regular dispersion on their surface promoting high enhancement factors (EF) and reproducibility. Besides, the TiO_2_ photocatalytic properties enable the substrate’s self-cleaning property for recyclability. In this review, we discuss the different methodological strategies that have been tested to achieve a high performance of the SERS substrates based on TiO_2_ NTs as solid support for the three main noble metal nanoparticles mainly studied for this purpose: Ag, Au, and Pt.

## 1. Introduction

Surface-enhanced Raman spectroscopy (SERS) has become one of the most attractive analytical techniques due to its versatility and high sensitivity for a variety of analytes like dyes, food additives, pesticides, explosives, DNA, and other biomolecules at very low concentrations [1]. Furthermore, SERS analysis presents a broad range of advantages: it is non-destructive, portable, easy to perform, highly sensitive, fast, cost-effective, and can be used with aqueous samples since the water background signal is negligible [2,3].

SERS measurements can be carried out in solution, by dispersing the analyte molecules in a colloidal metallic solution, or in a solid dry state, by adsorption of the molecules of interest on a SERS substrate. SERS substrates may consist of metal nanoparticles, roughened metallic surfaces, and more recently due to the advances of nanofabrication techniques, of nanoengineered surfaces with metallic nanoparticles deposited on particularly solid support [4,5].

SERS is a technique that couples Raman scattering from an organic molecule with surface plasmon resonance of a metal nanoparticle (MNP) in order to enhance the Raman-active vibrational modes of that molecule. This coupling allows amplifying the Raman signals with enhancement factors (EF) up to 10^10^ to 10^11^ [4,6,7,8]. However, some important aspects in SERS, besides EF, such as reproducibility, stability, detection limit, and selectivity have to be taken into consideration when assessing SERS performance. Among them, reproducibility is a major issue in SERS performance and it mainly relies on the availability of hot spots evenly distributed and is highly stable. Therefore, this technological development is aiming at the immobilization of MNPs in order to achieve this goal [3,4,9,10].

EF is highly dependent on the hot spots which are sites where the intensity of the Raman signal is enhanced. Hot spots can be localized in single nanoparticles with irregular shapes and sharp corners, or gaps between them. In terms of spherical nanoparticles, hot spots will be found in the interparticle spaces. Thus, in order to control hot spot distribution, it is desirable to anchor metal nanoparticles onto a solid substrate so they can be placed together in close proximity assuring stability and large enhancements [10,11,12].

Therefore, solid supports like mesoporous silica, polymers, alumina, and semiconductors have been studied in the last few years. Among them, semiconductors are promising solid substrates because they are chemically stable, reproducible and their morphology can be tuned, being able to adjust their surface properties to allow the periodic distribution of metallic nanoparticles and homogeneous enhancements across the surface [7,13,14,15].

Among semiconductors, titanium dioxide (TiO_2_) is one of the most investigated metal oxides for SERS because it enhances light scattering, it is biocompatible, and it shows self-cleaning performance through photocatalysis of adsorbed analytes, achieving the desired recyclability [13,16,17,18,19].

Titania nanotubes grown by anodization of Ti plates are of special interest because they may provide a higher surface area than titania nanoparticles deposited on a solid substrate. Besides, their electrical conductivity is higher which enables them to be used in electrocatalytic and photocatalytic processes that can be used for self-cleaning of SERS substrates. Moreover, their method of preparation allows synthesizing nanotube arrays with different dimensions and morphologies in a controlled manner, making it possible to obtain nanotubes of different sizes, diameters, and wall thicknesses that can be evaluated for SERS applications [20,21,22]. Furthermore, the crystal structure of TiO_2_ can be doped with different metallic and non-metallic ions (Cu^2+^, Co^2+^, Fe^2+^/^3+^, N^3−^…), affecting the band gap and the optical properties of the substrate, which may also impact the SERS activity [23,24,25].

We envisage the utilization of these nanostructured SERS platforms to enhance the sensitivity of Raman spectroscopy and be able to detect molecules of interest that are present at very low concentrations or even single molecules [26]. Due to a large number of applications of Raman spectroscopy, the availability of a robust, sensitive, reusable nano platform to perform SERS becomes attractive to a variety of fields. As such, it allows the detection of pathogenic entities in contaminated water [27], of prohibited drugs in urine [28], of contaminants in soils or water [29], and of cancer-related biomarkers in saliva or urine [30], among others.

This review aims to be a comprehensive, critical, and accessible review of general interest to the chemistry community because it discusses the state of the art of the use of titania nanotubes prepared by anodization as substrates for the main metal plasmonic nanoparticles (Ag, Pt, Au) currently utilized in SERS. Copper nanoparticles have been excluded from this review since no research has been reported in synergy with titania nanotubes for SERS applications.

The goals of the review are to:

(1) Provide a background on the SERS principles that pave the way to further discussion within the review.
(2) Justify the use of titania nanotubes prepared by anodization as an appropriate substrate for metal nanoparticles in SERS.
(3) Discuss the main features of the synthesis of titania nanotubes that influence the metal nanoparticle deposition.
(4) Present information about the metal deposition techniques on the TiO_2_ nanotubes substrate and the resulting SERS performance.

This review will focus on metal plasmonic nanoparticles of spherical shape deposited on titania nanotube substrates and the advantages of using TiO_2_ as a substrate for adequate nanoparticle deposition and dispersion, and also for self-cleaning (through photocatalysis). It will not cover other metallic nanoparticles morphologies such as cubes, flowers, rods, etc. The years of literature covered in this manuscript are mainly from 2015 up to date.

## 2. Raman Spectroscopy

Spectroscopy refers to the interaction of electromagnetic radiation with matter, the study of this interaction as a function of the wavelength of radiation provides insight into the composition of the sample, its crystalline structure, and even its electronic properties. Depending on the type of radiation used to excite the sample, different processes can be activated such as electronic or vibrational transitions. Radiation in the visible range activates molecular or crystalline vibrations; when monochromatic light irradiates a sample (photon energy Eo), two types of light scattering can result, elastic and inelastic. Elastic scattering (Rayleigh scattering) refers to the interaction of light with the sample upon which the net exchange of energy is zero, the energy of the incident and scattered photons is the same (E = Eo). During inelastic scattering events: (1) an electron is excited from the valence energy band to the conduction energy band by absorbing a photon; (2) the excited electron is scattered when a molecular vibration (vibrational energy Ev) is activated, and (3) the electron relaxes back to the valence band by emitting a photon with energy different form Eo (E = Eo ± Ev) [31,32]. The inelastic scattering of light is known as the Raman effect. However, it is a process that occurs in a small fraction of the scattered light (approximately 1 in 10^7^ scattering events) [33]. When the energy of the emitted photon is smaller by Ev, the energy of the molecular vibration, than that of the incident photon (E = Eo − Ev), the phenomenon is called Stokes Raman scattering. If the interaction causes the light photon to gain vibrational energy because the molecule was already in an excited state, then the energy of the scattered photon will be higher than that of the incident photon (E = Eo + Ev) and the process is called anti-Stokes Raman scattering, this phenomenon is less frequent than the Stokes counterpart [32]. Not all vibrations and rotations of molecules are evidenced in Raman experiments, the molecular vibration must induce a change in the molecular polarizability in order to be Raman-active and the intensity of the Raman scattering is proportional to the magnitude of the change in the molecular polarization [34]. By measuring the intensity of the scattered light as a function of the change of energy (frequency downshift measured in cm^−1^) of the scattered light we obtain a plot representing information about the allowed molecular vibrations of a molecule or crystal, the Raman spectrum [31]. Regarding instrumentation, the undisputable choice as a source of monochromatic light is a laser, micro-Raman spectrometers (coupled to optical microscopes) rely on different types (and hence wavelengths) of lasers to probe the sample, induce, and measure the Raman effect. There are numerous advantages to this technique: it is non-destructive; fast; highly sensitive; it does not require sample preparation; it can be performed on liquid, solid, or gaseous samples; it can explore aqueous solutions; the signal intensity is proportional to the species concentration thus it provides quantitative data; it has the possibility of portable and remote measurements using optical fibers [35]. However, despite the long list of advantages of Raman spectroscopy, there are downsides to the technique: fluorescence, laser-induced damage, and sensitivity; regarding the latter, as mentioned above, inelastic scattering happens in a very small fraction of the scattering events, thus the scarcity of non-elastically scattered photons requires sophisticated and expensive instrumentation for detection in order to achieve high sensitivity [35]. To overcome this sensitivity issue, variations of Raman spectroscopy have been introduced: RRS (resonance Raman spectroscopy), SERS (surface-enhanced Raman spectroscopy), and TERS (tip-enhanced Raman spectroscopy), among others [35]. In particular SERS, due to its inherent marriage to nanotechnology and to the achievement of improved sensitivity, has attracted a lot of attention and is the focus of numerous ongoing investigations. The principles of the technique are described below.

## 3. Surface-Enhanced Raman Spectroscopy (SERS)

In the late 1970s, it was discovered that Raman signals could be highly amplified by using a roughened silver substrate [36]. This new technique was named surface-enhanced Raman spectroscopy (SERS) and has attracted the attention of researchers because it is able to detect a wide variety of analytes like dyes, food additives, pesticides, explosives, DNA, and other biomolecules in very low concentrations [1]. Furthermore, SERS analysis possesses a broad range of properties: it is non-destructive, portable, easy to perform, highly sensitive, fast, cost-effective, and can be used when samples are present in water since the background signal is negligible [2,3].

In SERS, the amplification of Raman signals can be estimated with the enhancement factor (*EF*). The EF compares the ratio of the intensity of the strongest band in Raman spectroscopy (*I_RS_*) to that in SERS (*I_SERS_*), under identical experimental conditions (light source, light power, time of acquisition, etc.). It also considers the ratio of the number of adsorbed molecules in a given scattering volume for SERS (*N_SERS_*) and Raman spectroscopy (*N_RS_*) analysis respectively, as shown in the next equation [6,37].

(1) $$EF=\frac{I_{SERS}/N_{SERS}}{I_{RS}/N_{RS}}$$

The previous equation is representative for each substrate but sometimes in analytical applications, one desires only to know how much the signal is amplified. Another parameter, the analytical enhancement factor (*AEF*) can be calculated, where the number of adsorbed molecules is replaced with the concentration of the analyte in the sample for Raman spectroscopy (*C_RS_*) and SERS (*C_SERS_*), respectively as written in Equation (2). This value, although useful for analytical applications cannot be used to compare different substrates because not all the molecules in the sample will contribute to the signal, only the ones close to the substrate [6,37].

(2) $$AEF=\frac{I_{SERS}/C_{SERS}}{I_{RS}/C_{RS}}$$

In order to understand how SERS works, it is imperative to remember, as mentioned above, that the intensity of the Raman scattering is proportional to the magnitude of the net change in the molecular polarization and that this affects the induced dipole of the molecule *μ*, since it is expressed as the product of the polarizability *α*, and the strength of the electric field *E* (Equation (3)).

(3) μ=αE

Thus, for achieving stronger Raman signals, one of both parameters can be increased, which is possible by two mechanisms: (i) electromagnetic and (ii) chemical [1,6,9]. The electromagnetic mechanism is believed to be the most important and arises due to the localized surface plasmon resonance (LSPR) of a nanostructured metallic substrate, an optical phenomenon in which light interacting with noble metallic nanoparticles, smaller than the incident wavelength, provokes the surface electrons to become polarized and oscillate collectively, which is known as a surface plasmon. During this process, the electrons will be displaced from the nuclei and then return to the original position, due to the Coulombic attraction, producing an oscillation at a particular frequency [38,39,40]. Then, when the incident photons are in resonance with this oscillation, a strong electric field will be produced [21]. Electromagnetic enhancement relies on Raman-active molecules being confined within these electromagnetic fields [41].

The oscillation resonance frequency is dependent on the nature of the metal, size, shape, and surrounding medium; when resonance is achieved with the incident light, the Raman signal can be amplified with an EF up to 10^10^ or 10^11^, allowing the detection of single molecules. For silver, gold, and copper nanoparticles with sizes between 30 and 100 nanometers (nm), the plasmon resonance falls within the visible light range, presenting collective oscillations on rough substrates with large surface areas [4,6,7,8].

**Figure 1:**
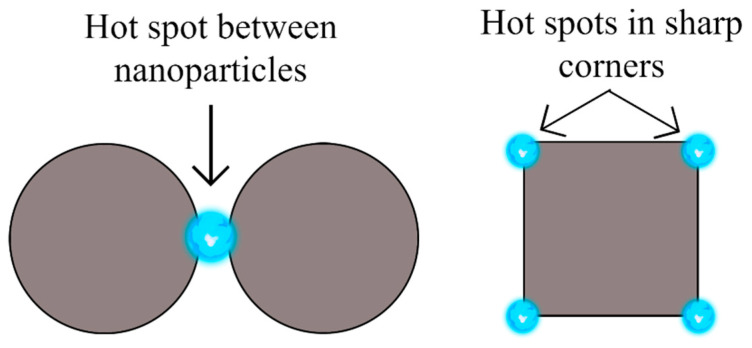
Location of hot spots.

However, the generated electric field is not uniformly distributed throughout the substrate. Instead, it is localized in narrow regions known as hot spots which can be regions over the surface of single nanoparticles with irregular shapes and sharp corners, or gaps between neighbor nanoparticles, which are responsible for larger intensities [42]. The subsequent diagram in Figure 1, is a visual representation of how hot spots are located within nanoparticles.

Consequently, moderate EFs are more frequent, because it is much more possible that the analytes are somewhere on the surface of the substrate rather than in between nanoparticles in hot spot regions [43]. This has a direct impact on the reproducibility of SERS because the number of nanoparticles can vary greatly from one place to another and those substrates with non-homogeneous coverage will produce signals with different enhancements, depending on the position where the measurement takes place; areas where hot spots are found will produce large enhancements, while regions with single nanoparticles will not [10,11]. Hence, substrates with high EF values are, in most cases, not reproducible. In fact, the usual relative standard deviation (RSD) of SERS measurements is between 15 and 20%, and when the system is averaged as a whole, the enhancement factor rarely exceeds 10^7^ or 10^8^ [4,12]. It is due to the location of hot spots that large enhancements are rare.

For this reason, even though the enhancement factor is important, it should not be seen as crucial for defining the performance of a SERS array. Aspects such as reproducibility, stability, detection limit, and selectivity have to be taken into consideration as well. As such, it becomes necessary to engineer nanostructured substrates with a greater number of hot spots arranged periodically to provide homogeneous enhancements. One strategy is to reduce the gap between nanoparticles and many reports claim that aggregation is desirable, but this should be evaluated carefully because it is difficult to control nanoparticle aggregation [3,4,9,10,43].

The chemical mechanism is based on the formation of a charge-transfer complex between the analyte and the metallic surface. When these two are close enough, their wave functions will overlap and electrons can be transferred, changing the polarizability of the molecule and intensifying the Raman signals. For this reason, the enhancement will decrease rapidly as the distance between the analyte and the substrate increases, but they do not have to be in direct contact [1,43,44]. However, this mechanism alone does not result in SERS, because its maximum contribution to the EF is of 2–3 orders of magnitude. It is the synergy between both mechanisms (electromagnetic and chemical) that makes it possible to obtain amplified Raman signals [1,7,44].

To sum up, for obtaining good SERS performance, it should be remembered that SERS is wavelength dependent and it is imperative to use an appropriate light source that is in resonance with the surface plasmons of the metallic nanostructures [9]. Other aspects that have to be considered are the optical, morphological, and adsorption properties of the substrate and its affinity to the analyte molecule [6,45].

## 4. SERS Substrates and TiO_2_/MNPs Arrays

The substrate is the most important factor to take into consideration when performing SERS experiments because its components, shape, size, and interparticle spacing will have a great influence on its performance; hence, the morphological characteristics should be carefully studied in order to assure large enhancements. Additionally, several requirements must be met including [4,12,46]:

(1) Reproducibility, providing similar enhancements across the whole surface by arranging metal nanoparticles regularly. In the same sense, the fabrication of SERS substrate must be reproducible from array to array or from batch to batch.
(2) Stable and unresponsive to environmental conditions like humidity, oxygen, and light.
(3) Cost-effective and easy to handle.
(4) Highly stable and biocompatible if biological molecules or specimens are to be detected.

In general, when noble metals are used as substrates for SERS applications, they can be found as nanoparticles in suspension or nanostructures deposited on a surface. Silver and gold are the most common SERS substrates because of their LSPR effect in visible light and the high SERS enhancements arising from the control in their size and shape, but nanoparticles in suspension tend to aggregate, which changes the interparticle array. This is an unstable and non-reproducible system, and the use of stabilizers is inappropriate because they could interfere with the signals [6,12,46,47].

Secondly, when metal nanoparticles (MNPs) are attached to a solid support, they can be placed together in close proximity assuring stability and large enhancements. Thus, nanostructured semiconductors or insulators are used to support plasmonic metals, even paper-based SERS substrates are available [5]. However, one disadvantage of a typical matrix of deposited MNPs is that they cannot be easily reused because the analytes will stay adsorbed on the surface and cause interference. This is not profitable from the economical point of view; therefore there is an increasing interest in fabricating novel hybrid SERS substrate nanoplatforms, that allow achieving not only maximum enhancement and reproducibility but recyclability as well [46,48,49].

Thereafter, materials like quantum dots, graphene, and semiconductors have been studied in the last few years. Among them, semiconductors are promising SERS supports because they are resistant to degradation, reproducible, and their geometries can be controlled, being able to adjust the surface properties to allow the periodic distribution of metallic nanoparticles and homogeneous enhancements across the surface. Some of the nanostructured semiconductors that have shown good SERS performance are silicon nanowires and nanostructured ZnO, ZnS, InAs, Pb_3_O_4_, CuO, CdTe, NiO, and TiO_2_ [4,7,13,14,15].

It is important to point out that semiconductors are able to show SERS performance due to the chemical mechanism and research has been done to use them as SERS substrates. Since they do not present electromagnetic enhancement, their EF is expected to be much lower than that of silver or gold metals [7,18]. Table 1 presents a comparison of some studied semiconductors for SERS performance.

Titanium dioxide (TiO_2_) is one of the most investigated semiconductors for SERS because it enhances light scattering, it is biocompatible and it shows self-cleaning performance which means that it can degrade adsorbed analytes by photocatalysis, achieving the desired recyclability. Moreover, it is synthesized in different morphologies, including nanoparticles, nanorods, and nanotubes, with outstanding properties such as relatively high conductivity, corrosion resistance, and stability, making them appropriate for diverse applications ranging from photoelectrocatalytic degradation of pollutants to dye-sensitized solar cells [13,16,17,18,19].

**Figure 2:**
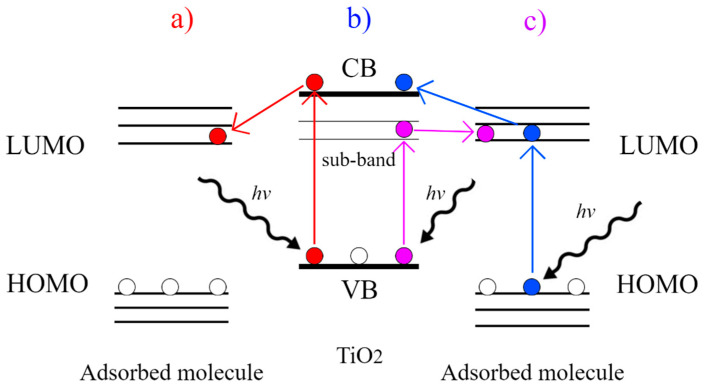
Charge-transfer mechanisms between TiO_2_ and adsorbed molecules.

TiO_2_ shows SERS enhancement due to the chemical mechanism, arising from the charge transfer with the adsorbed molecules, a phenomenon that can occur by different paths as shown in Figure 2: (a) Upon irradiation with light of sufficient energy, electrons (in red) in the valence band (VB) of TiO_2_ are excited to the conduction band (CB) and then transferred to the lowest unoccupied molecular orbital (LUMO) of the adsorbed molecule; (b) electrons (in blue) in the highest occupied molecular orbital (HOMO) of the molecule are photoexcited to the LUMO and then transferred to the CB of TiO_2_; (c) electrons (in purple) in the VB of TiO_2_ are excited by photons to a sub-band energy level and then transferred to the LUMO of the adsorbed molecule [61,62].

**Figure 3:**
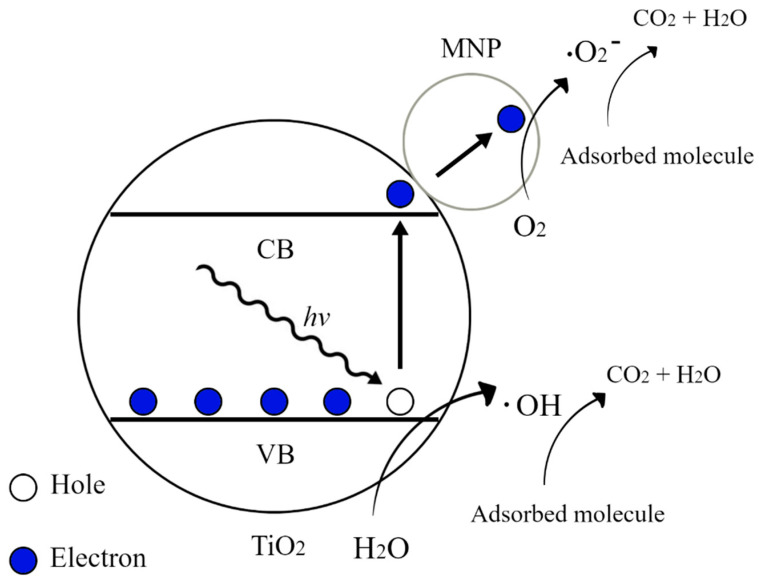
Phototatalytic mechanism of a TiO_2_/MNP substrate to degrade adsorbed molecules.

Even though the SERS activity of semiconductors is usually poor, one of the main advantages of using TiO_2_ as a support to deposit metal nanoparticles is that it allows, through the photocatalytic process, the self-cleaning of the substrate. In the photocatalytic mechanism, when irradiated with light of sufficient energy, the electrons (e^−^) of titanium dioxide are excited from the valence band to the conduction band and are trapped by the metallic nanoparticles (MNPs) leaving holes (h+) behind in the VB of TiO_2_. After this, the holes react with water to produce the hydroxyl radical (OH·), while the electrons react with molecular oxygen to form the superoxide radical (O_2_^−^), both of which can undergo further reactions to produce species that are powerful oxidants and can effectively degrade organic molecules [63,64]. The process is represented in Figure 3.

Among the TiO_2_ nanostructures that have been studied as supports for MNPs, nanotube arrays prepared by anodization have shown promising properties to build SERS platforms. Several features make them appropriate for this purpose: (a) they can provide an appropriate acidic surface for the MNPs deposition [20]; (b) the high surface area of TiO_2_ nanotubes allows to deposit a great number of MNPs, being able to have many hot spots; (c) due to their photocatalytic properties can degrade the adsorbed molecules upon light irradiation to use the substrate for next measurements [65]; (d) when the analytes are adsorbed on bare titanium dioxide nanotubes, no signal is observed, except for the band corresponding to titanium dioxide, as it has been reported by several works [20,21,66].

### Titanium Dioxide Nanotubes (TiO_2_ NTs) Prepared by Anodization

According to Ling and co-workers, both the metallic nanoparticles and the nanotubes have a direct impact on the enhancement [66]. Certainly, the knowledge of how the different dimensions of the nanotubes will affect the distribution of the metallic deposit; and consequently, the resulting SERS enhancement and reproducibility, is of prime importance to synthesize nanotube arrays with proper features.

Essentially, the anodic oxidation method allows preparing nanotubes with different dimensions in a controlled manner by varying synthesis conditions like applied voltage, anodization time, electrolyte composition (fluoride, pH, water, and organic solvent content), and temperature; making it possible to obtain nanotubes of different sizes, diameter, and wall thickness that can be evaluated for SERS applications.

The TiO_2_ NTs formation mechanism by anodization and the effect of the change in their synthesis parameters has been thoroughly discussed by academia [67,68,69,70,71,72,73]. These parameters can be adjusted to achieve the desired morphology and dimensions from these nanostructures. For instance, high water content limits the nanotube length growth and therefore an organic modifier is added to aid the formation of longer nanotubes. In addition, as a higher voltage is applied, bigger nanotube diameters can be achieved. Anodization time is also important as it allows, to some extent, the nanotubes’ growth.

While no single mechanism has been universally confirmed, the most accepted and conventional theory is field-assisted dissolution (FAD). FAD theory is based on the fact that anodic nanotube growth mirrors the growth mechanism of porous nanomaterials such as porous anodic alumina (PAA) [67]. As a result, self-organized titania growth is dictated by the three reactions; the electrochemical oxidation of Ti into TiO_2_, the electrical field-induced chemical dissolution of TiO_2_, and the F^−^ induced dissolution of TiO_2_. This means an external field induces a chemical reaction that forms self-organized nanotubes on top of an oxide layer [68]. To further understand this, it is important to know the chemical reactions that happen through the anodization process and regulate the formation of anodic TiO_2_ nanotubes in the electrode. As it was previously mentioned, the first of this reaction is the oxidation of Ti by reacting with the oxygen molecules in the solution, which forms an oxide layer (reaction 1).

(4) $$Ti^{4+}+2H_{2}O\to TiO_{2}+4H^{+}$$

**Figure 4:**
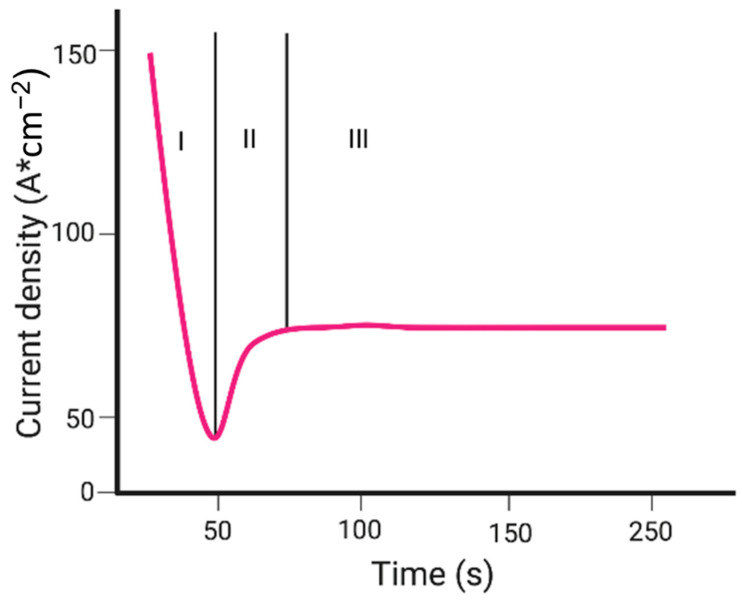
Representative current–time curve for the formation of TiO_2_ by anodization using F^−^ electrolytes under a constant voltage. The curve is divided into 3 phases. Phase I is characterized by a noticeable current decay. Phase II raises the current under a time lag, and phase III reaches a constant steady current.

Because the system is under constant voltage, the field of the oxide layer is reduced, which increases oxide thickness and sharply decreases its current density, this is known as phase I. The sudden drop in current can be seen in Figure 4. As the nanotube continues to grow, internal stress and volume expansion produce cracks causing an uneven distribution of the electric field [69]. Additionally, the dissolution of the oxide layer is chemically attacked by F^−^ ions [70] found on the electrolyte. This reaction happens in the center of the local electric field, which forms a sort of void embryo and deepens each local hole [69]. This process forms water-soluble [TiF_6_]^2−^ complexes that also prevent the precipitation of Ti(OH)_x_O_y_ as more arriving Ti^4+^ ions enter the oxide/electrolyte interface [68]. The migration of F^−^ ions towards the oxide/electrode interface helps in the rate of the oxide dissolution and formation of complexes and depends on the concentration, the field strength, and pH drop at the surface [71]. The oxide dissolution process can be described by:

(5) $$TiO_{2}+6F^{-}+4H^{+}\to {\left[TiF_{6}\right]}^{2-}+2H_{2}O$$

The formation of [TiF_6_]^2−^ by Ti^4+^ ions can be described by:

(6) $$Ti^{4+}+6F^{-}\to {\left[TiF_{6}\right]}^{2-}$$

**Figure 5:**
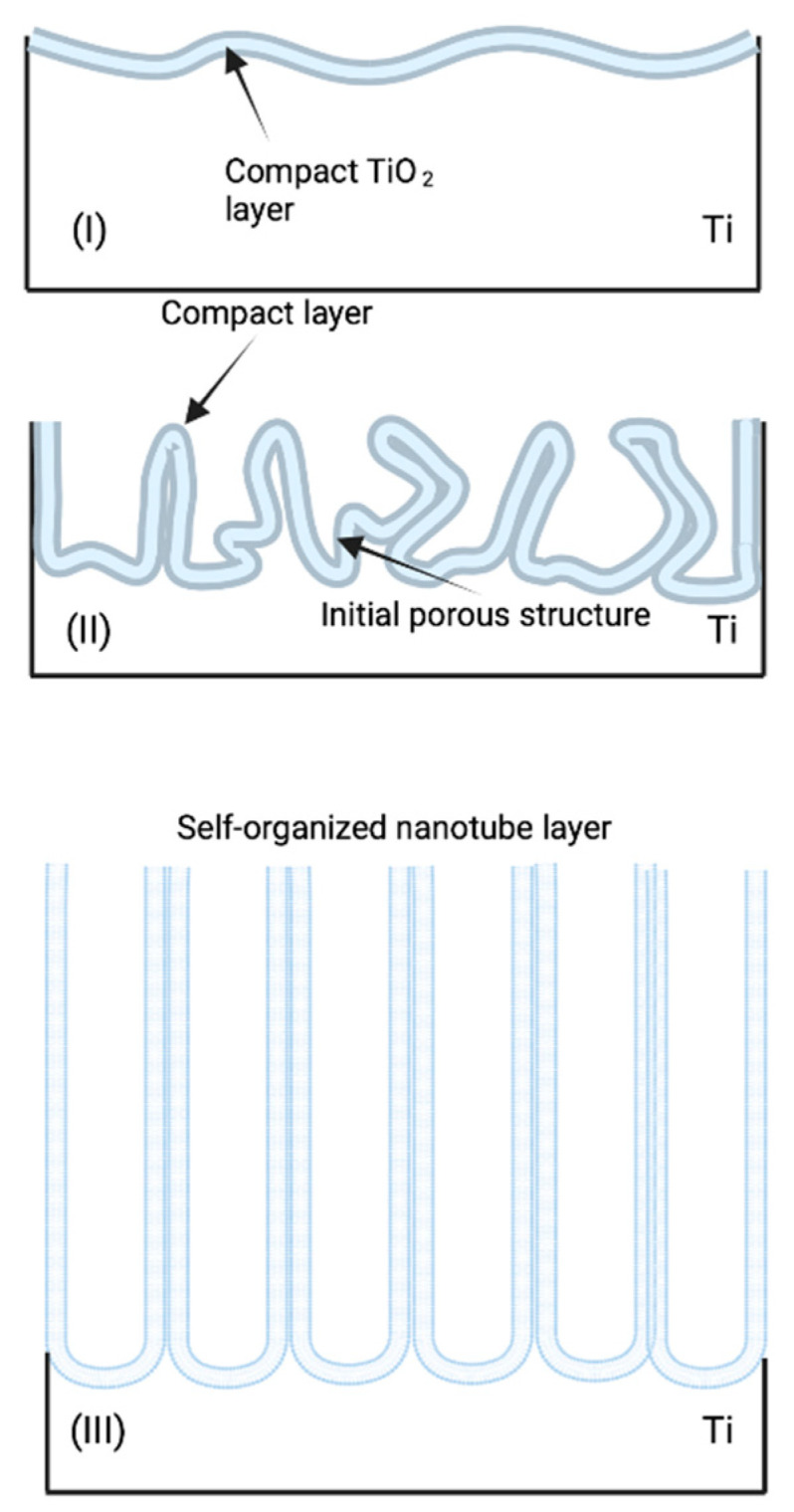
Formation process of TiO_2_ by anodization. The process is divided into 3 phases. Phase I consists of the formation of a compact barrier oxide. Phase II consists of the creation of pores after the surface activation by the oxide dissolution. In phase III, the layer of thinner self-organized nanotubes is formed.

The two previous chemical reactions locally activate the surface. As a result, random pore growth in the surface is able to increase the active area, which increases the current density [72] and induces a tree-like growth of the pores. This increase in surface area induces a time lag in the rise of the current. The shorter this lag is, the higher the fluoride concentration will be. This second part is known as phase II. The third and final phase happens when the oxide dissolution rate and the oxide growth rates (reactions 2 and 1 respectively) reach a chemical equilibrium [73]. This causes the current density to be in a steady state, and the tubes on the top have thinner walls than their bottom counterparts, which are more closely packed [68]. The result from this is long, thin self-organized TiO_2_ NT arrays. The entire forming process of TiO_2_ NT arrays can be seen in Figure 5 with each respective phase.

Nanotube diameters usually range from a few dozens to hundreds of nanometers, and the length consists of a few micrometers. Consequently, research works that evaluate the impact of TiO_2_ different nanotube dimensions on SERS activity have emerged in order to guide the synthesis to achieve optimal performance [20,21,22].

Among them, we can mention the work of Pisarek and colleagues. They varied the anodization voltage from 5 to 30 volts (V) in an electrolyte consisting of 0.86 wt.% NH_4_F, 47.14 wt.% deionized (DI) water, and 52 wt.% glycerol, and using pyridine as a probe molecule for SERS (anodization time was not reported). The SEM images revealed that the nanotubes were about 1 μm in length and that the diameter increased from 20 nm at an applied voltage of 5 V to 120 nm at 30 V. All the samples were annealed at 650 °C for 3 h, obtaining a mixture of anatase and rutile crystalline phases. Then, they covered the nanotubes with silver nanoparticles (Ag NPs) using a sputtering deposition technique, obtaining nanoparticles with a diameter below 100 nm. As a result, they observed that the nanoparticles were located inside the nanotubes anodized at 25 V, whereas the nanoparticles completely covered the surface of the nanotubes anodized at 10 V. The latter had the largest enhancements because there was a higher number of SERS active sites [21].

They also found that the position and the ratio of the SERS signals for pyridine remained unchanged for the nanotubes with different diameters; but, it was the signal intensity that was influenced by the dimensions of the nanotubes. The authors concluded that the EF depends on the way that silver nanoparticles are distributed onto the nanotubes [21]. In fact, Roguska and teammates suggest that it is not possible to obtain equivalent silver deposits on nanotubes with different dimensions because the diameter will greatly influence the way the nanoparticles are distributed onto the nanotubes [74].

For their part, Ling and co-workers prepared TiO_2_ NTs by anodization in an ethylene glycol electrolyte containing 0.5 vol% HF, for 15 min and applying 20, 30, 40, 50, and 60 V for obtaining nanotubes of 40, 60, 80, and 100 nm, respectively (the diameter of the nanotubes anodized at 60 V was not reported). Next, the samples were annealed in air at 500 °C for 2 h to obtain the anatase phase. Then, 0.03 mg/cm^2^ of Ag NPs were deposited by vacuum plasma sputtering for 10 min under a pressure of 6 Pa, and kept in humid air for aging, and were exposed to UV light. The nanoparticles were about 50 nm in size and their deposition changed depending on the nanotube diameter. The nanotubes prepared at 20 V had a small diameter and the nanoparticles could not get in, so they were located only at the top, but as the diameter of the nanotubes grew bigger the nanoparticles began to migrate to the interior. These differences were perceived in the SERS experiments where rhodamine (R6G) was used as a probe molecule. It was observed that the SERS signal was not proportional to the nanotube diameter and higher intensities were obtained for the nanotubes anodized at 30 and 60 V. The nanotubes anodized at 30 V had a diameter of 60 nm and there was a large number of silver nanoparticles deposited that increased the roughness of the surface. Regarding the nanotubes anodized at 40 and 50 V, the nanoparticles were relatively isolated from one another, since they were on the inside of the nanotubes; with an anodization of 60 V, the nanoparticles were closer to one another, providing narrow slits between particles [66].

On their side, Chen and co-workers prepared nanotubes using different anodization parameters. In the first experiment, the electrolyte consisted of ethylene glycol with 0.25 wt.% NH4F and 2 wt.% DI water; the anodization was performed at 60 V for 30 min. The nanotubes were annealed at 450 °C for 2 h to obtain the anatase phase. Followingly, gold nanoparticles (Au NPs) were electrodeposited by pulse current using a two-electrode configuration where the TiO_2_ NTs sample was the working electrode and a Pt plate the counter electrode, in a solution of 1 mM HAuCl_4_ and 0.1 M H_2_SO_4_. The applied current was alternated between 0.1 s on, applying 10 mA/cm^2^, and 0.3 s off, for a period of 40 s. All of this resulted in a surface layer of TiO_2_ with pores of 60 nm in average diameter and 7 μm in length, but there was no gap between each pore; thus, the surface was continuous. The nanoparticles, with sizes ranging from 40 to 140 nm, were located at the top of the TiO_2_ and no nanoparticles were found on the inside of the pores [20].

Secondly, other nanotubes were prepared in a glycerol and distilled water electrolyte (*v/v* = 10) with 0.5 M H_3_PO_4_ and 0.2 M NH_4_F, applying 30 V for 2 h. The annealing and Au NP deposition were the same as described previously. In this case, a tubular morphology was observed, having gaps between the nanotubes that were 130 nm in diameter and 1.3 μm in length In this case, the TiO_2_ layer presented a tubular morphology, having nanotubes separated from one another by gaps; hence, the surface was discontinuous, and the nanotube diameter was about 130 nm and the length about 1.3 μm. This structure allowed the nanoparticles, with sizes from 80 to 120 nm, to be deposited on the open ends and in the gaps between nanotubes, being closer to one another. SERS measurements on 4-mercaptobenzoic acid (4-MBA) showed that this structure provided twice the intensities as the nanocomposite mentioned earlier, because by having nanoparticles in the gaps between each nanotube, it was possible to shorten the distance between them, generating hot spots with strong electric fields. Thus, it was demonstrated that by having a similar amount of metal deposit, the structure of the TiO_2_ surface will play a key role in the SERS activity [20]. As a result, TiO_2_ NTs are promising supports because, by controlling the geometry upon conditioning anodization parameters, it is possible to have metallic nanoparticles deposited in the gaps between each tube to provide a large number of hot spots as well as a homogeneous surface coverage [11,75].

As far as studying an optimal wall thickness for SERS performance, little attention has been paid to this feature, but the work of Sun and collaborators has explored this parameter. They performed FDTD (finite-difference time-domain) simulations with a model of titania nanotubes closely packed in two dimensions, in which nanotubes adopted a hexagonal arrangement with a diameter of 60 nm and a wall thickness from 17 to 23 nm. In this model, the size of Ag nanoparticles is determined by the NTs wall thickness. They used a polarized 514 nm plane wave that was placed above the Ag-coated TNTs and polarized to the *x*-axis and whose injection direction was –*z*; and the electromagnetic field was recorded by a frequency domain field profile monitor in the *x*–*z* plane. From this simulation, they found that the maximum electromagnetic field is achieved with a 20 nm wall thickness [76].

On the other hand, it can be seen that all reports anneal the anodized nanotubes at different temperatures in order to convert them from amorphous to crystalline phases and improve their mechanical stability as well as their adhesion to the Ti substrate [66,74]. There are three naturally occurring crystalline phases for titanium dioxide: anatase, rutile, and brookite. It is reported that the anatase phase begins to transform to rutile at 600 °C, but this value can fall in the range 400–1200 °C depending on the processing method [77].

In terms of appropriate crystalline phases for SERS applications, it has been indicated that a mixture of anatase and rutile might help in ameliorating the activity [78,79,80]. So, in order to compare different crystalline phases in SERS experiments, Pisarek and collaborators annealed the as-prepared TiO_2_ NTs at 450 °C and 650 °C for 3 h. For the lower temperature, the anatase phase was obtained, which was covered with a silver deposit of 0.015 mg/cm^2^; by annealing the nanotubes at 650 °C a mixture of anatase and rutile phases were obtained, which were covered with 0.010 mg/cm^2^ silver deposit. SERS measurements on pyridine revealed that the position and shape of the signals did not change but the anatase Ag NPs/TiO_2_ NTs composite had less intense signals than the anatase/rutile mixture [21].

Notably, the different atomic arrangements of rutile and anatase can have an impact on the way that TiO_2_ and Ag NPs interact with each other [81]. In concrete, Huang and collaborators compared Ag NPs deposited on anatase and rutile TiO_2_ nanoparticles. It was found that silver was less abundant on anatase and, consequently, their SERS signals for R6G were weaker. On the contrary, the rutile substrate had more silver deposited, which allowed having a higher number of hot spots [82].

Finally, Yang and partners demonstrated that the enhancement factor on 4-MBA changes depending on the crystalline phase of titanium dioxide. They prepared TiO_2_ nanoparticles by a hydrothermal method and annealed the samples at different temperatures: 500, 550, 600, and 650 °C for 2 h. It was seen that when annealed at 400 and 450 °C only anatase crystalline phase was present but when the temperature began to increase, the amount of rutile content did as well. As a consequence, at 500, 550, and 600 °C a mixture of anatase/rutile is obtained, and at 650 °C the crystalline phase is mostly rutile. However, it must be kept in mind that when the temperature rises, the crystallite size increases, which reduces the surface area and the number of analytes that can be adsorbed, affecting the SERS signals. Therefore, the samples annealed at 550 °C gave the strongest intensities, explained by the interaction between both crystalline phases, anatase, and rutile that, at an appropriate ratio (85:15, respectively), are able to separate charge carriers; and thus, enhance the charge transfer between TiO_2_ and the adsorbed molecule [80,83]. This annealing temperature was optimal because at temperatures higher than 550 °C not only is the surface area decreased but the number of surface defects (oxygen vacancies) does as well since the anatase phase is lowered as rutile increases. This has a detrimental impact on the SERS performance because oxygen vacancies are able to trap the charged species and hinder the recombination [80,84]. Therefore, by controlling the ratio of anatase and rutile within the TiO_2_ matrix, it is possible to enhance the SERS signal due to a separation of the charge carriers, promoting the chemical mechanism [80,85].

Furthermore, it has been reported that the charge separation by the anatase/rutile mixture is helpful in terms of photocatalytic activity [86]. As an example, Li and teammates prepared nanosized TiO_2_ via the acidic hydrothermal method and, upon varying the amount of added tartaric acid to the system, they were able to obtain different anatase/rutile ratios. They found out that a mixture of 77% anatase and 23% rutile provided the highest photocatalytic activity towards the degradation of rhodamine B (RhB) and methyl orange; the degradation was even higher than in pure anatase or rutile phases, which is attributed to the separation of the electron-hole pair provided by the anatase/rutile mixture [87].

Similar results were obtained by Li and partners by showing that a mixture of 97% anatase and 3% rutile in TiO_2_ nanofibers is more efficient towards the photocatalytic degradation of RhB than pure anatase or pure rutile phases. Accordingly, electrons will be transferred from anatase, with a band gap of 3.2 eV, to the lower energy conduction band of rutile, whose band gap is 3.0 eV [88]. Thus, rutile acts as an electron trap, which increases the lifetime of the photogenerated electron-hole species, and hence, can serve for the photocatalytic process [87,88].

## 5. Noble Metal Nanoparticles

### 5.1. Silver Nanoparticles (Ag NPs)

For SERS applications, it was mentioned that metallic surfaces are required, copper, gold, and silver are mainly used; however, gold and silver are more stable than copper under ambient conditions and, compared to gold, silver is able to give higher signal enhancements [89]. To this respect, Sun and colleagues reported an enhancement factor of 2.26 × 10^8^ with a Ag NPs/TiO_2_ NTs substrate to detect 2-mercaptobenzoxazole with a limit of detection close to 10^−^^9^ M [76]. On their part, Lamberti and his team reported a limit of detection (LOD) for RG6 in the range of 10^−^^14^ M, defining this value as the concentration of the analyte at which the signal is three times greater than the standard deviation of the blank signal [44,90]. What is more, silver nanoparticles are increasingly being used in optical sensors and in diagnostics due to their exceptional optical properties and because they represent a low-cost and scalable option for SERS purposes [91,92].

Silver nanoparticles are the most used substrates since they have a broad surface plasmon resonance, which goes from the visible to the near-infrared regions, and because they are stable and easy to prepare [93]. Various techniques are reported to obtain Ag NPs on solid substrates for SERS applications including thermal evaporation, ion sputtering, electrochemical, wet chemical, vapor-phase synthesis, and photoreduction [89,94].

#### Silver Nanoparticle Deposition on TiO_2_ NTs

Notably, wet chemical techniques have been widely used for obtaining Ag NPs of different shapes and sizes by using capping ligands like trisodium citrate and poly(vinylpyrrolidone) (PVP) but these species cause interference in SERS measurements. In contrast, by using photoreduction methods it is possible to obtain clean substrates that are free of impurities [93].

As an example, Lamberti and co-workers used energy-dispersive X-ray spectroscopy (EDX) to analyze the composition of TiO_2_ NTs (which were not annealed) before and after the deposition of Ag NPs by the photoreduction process. Firstly, it was possible to observe signals arising from titanium dioxide but also from fluorine and carbon, present in the electrolyte solution during the anodization process. Then, after silver deposition, which was done in a microfluidic chamber filled with a solution of silver nitrate in water and ethanol, and irradiated with UV light for 5 min, not only was a silver signal noticeable in the EDX spectra but the bands corresponding to carbon and fluorine were reduced. Another advantage of UV irradiation is that it heats the sample and partially crystallizes the nanotubes, as evidenced by XRD measurements where bands arose due to the anatase phase [48].

On the other hand, the sputtering deposition technique is also able to prepare clean substrates and, upon varying the synthesis conditions, it is possible to obtain nanoparticles of different size and size distribution [95,96]. In this sense, sputtering time can be modified to obtain nanoparticles of different sizes as mentioned by Rezaee and co-workers [97], and some reports have done this to study the effect on the SERS activity.

As an example, Zhang and collaborators [98] used the magnetron sputtering technique to deposit silver nanoparticles onto previously annealed TiO_2_-x NTs for different times: 20, 25, and 30 s (s). Upon SEM analysis, it was seen that with larger deposition times the nanoparticles grew bigger, which had an impact on the enhancement factor on RhB. The sample sputtered for 30 s had the largest enhancement, with a value of 2.47 × 10^5^, and a sputtering time of 20 s had the smallest, whose value was 2 × 10^5^, concluding that small and low-density Ag NPs are not optimum [98]. Thus, it is evident that SERS activity is dependent on the size of the nanoparticles but also on their aggregation state [93,99].

Additionally, it is possible to vary the amount of silver deposit onto titanium dioxide nanotubes to study the effect on the SERS signals, In fact, there are some reports in the literature where sputtering is used to deposit silver nanoparticles onto TiO_2_ NTs prepared by anodization. In several reports, the sputtering is performed under a pressure of 3 × 10^−^^3^ Pa and the silver deposit usually varies from 0.01 mg/cm^2^ to 0.2 mg/cm^2^, and it is mentioned that, generally, the signals for probe molecules such as pyridine or mercaptobenzoic acid, are either almost equal or more intense than those of a reference electrochemically-roughened silver substrate, whose enhancement factor is in the range 10^6^–10^7^, demonstrating that the superior activity makes these substrates promising for SERS applications [21,74,96,100].

In particular, Roguska and collaborators used sputtering to obtain different silver deposits onto TiO_2_ NTs with a diameter of 100 nm. By SEM images, it was seen that a surface coverage of 0.01 mg/cm^2^ resulted in nanoparticles relatively isolated from one another and with sizes ranging from 5 to 20 nm that were located at the top of the nanotubes and on the sidewalls; while a silver deposit of 0.09 mg/cm^2^ presented an agglomeration of the nanoparticles in the form of rings at the top of the nanotubes reducing their internal diameter, but single nanoparticles from 10 to 40 nm in size were present as well. Notably, the highest SERS signals for pyridine were achieved with the latter substrate because there was a considerably larger number of narrow slits between particles. Lastly, the authors showed that the signal could not be increased even further by having more silver deposits because by then, the whole surface would be covered with a thick silver layer [101].

Likewise, Pisarek and partners used evaporation in a low vacuum method to deposit different amounts (0.01, 0.02, and 0.03 mg/cm^2^) of Ag NPs onto TiO_2_ NTs with a diameter of 38 ± 9 nm. For the smallest amount of silver deposited, the nanoparticles were found on the top and side walls of the nanotubes, with some agglomerates found; the silver surface area increased with larger deposits, having agglomerates of nanoparticles with narrow slits between them, ranging from several to a few dozen nanometers. This, in turn, led to larger SERS signals, whose intensity increased with the amount of silver. For pyridine, p-mercaptobenzoic acid, and rhodamine dye, enhancement factors in the order of 10^6^ were achieved, being attributed to the surface area provided by the nanotubes and appropriate size distribution of the nanoparticles [22].

For their part, Xie and co-workers used an electroless deposition process in which TiO_2_ NTs were immersed in a solution 0.1 M of [Ag(NH_3_)_2_]OH for 5 min and then in 0.1 M glucose for 5 min. This was referred to as one cycle and they prepared samples by repeating the process up to five cycles. The size of the nanoparticles increased with the number of cycles and by the fifth cycle, the nanoparticles had irregularities in their size distribution, and the interparticle distance varied from several to tens of nanometers. As a result, the SERS signals for 4-mercaptobenzoic acid (PMBA) were higher for the substrate prepared with five cycles, because the interparticle distance was small, obtaining an AEF of 1.8 × 10^5^ [19]. For this reason, it has been mentioned that one can increment the enhancement factor upon increasing the particle size [99].

However, Lamberti and his group deposited Ag NPs onto previously annealed TiO_2_ NTs using direct current sputtering with a current of 40 milliamperes (mA) and with deposition times of 30, 60, and 90 s. By field emission scanning electron microcopy (FESEM), they found that the nanoparticles became larger with deposition time: For 30 s, the nanoparticles were 63 ± 15 nm in size and with interparticle distances varying from 5 to 10 nm to 20 to 50 nm; at 60 s of deposition, the equivalent diameter of the nanoparticles was 99 ± 10 nm, and at 90 s the particles began to coalesce among them to create thick clusters that covered the nanotubes. This had an impact on the SERS activity because the LOD of R6G changed from 10^−^^14^ M for a deposition time of 30 s, to 10^−^^8^ M for 90 s which can be explained by taking into account the specular reflectance measurements. The nanoparticles deposited for 30 s had a great distribution of sizes and interparticle gaps, thus having a broad resonance peak in the range 360–500 nm, being in resonance with the excitation source whose wavelength was 514.5 nm; with higher deposition times, there was a reduction of the plasmon resonance band as well as a blue shift down to 355 nm due to the weakening of the titanium dioxide substrate contribution. The authors concluded the low LOD is achievable because of the synergy between charge transfer and electromagnetic mechanism because, without the silver deposit, R6G in low concentrations could not be detected. Additionally, the best results were obtained when the excitation source matched the surface plasmon resonance of the substrate [44].

Therefore, it becomes evident that it is possible to modulate the optical properties of the metallic nanoparticles regarding the shape, position, and width of the localized surface plasmon resonance peak, by adjusting the synthesis conditions [102,103]. The reason being is that the LSPR peak is very sensitive to any change in the environment of the substrate, and by modifying the size or shape of the nanoparticles it is possible to shift it [103].

In fact, He and colleagues observed this phenomenon and used it to investigate how the SERS activity towards RG6 is affected by varying the size of Ag NPs and its LSPR peak. They deposited silver nanoparticles onto a functionalized glass substrate to obtain sizes from 35 to 65 nm, finding that the particles were either alone or formed clusters. In addition, as the particle diameter increased, the homogeneity of the size distribution decreased, as well as the surface coverage. Despite this, with UV-visible spectroscopy, it was seen that with larger nanoparticle size, an absorption band appeared above 600 nm that red-shifted gradually, arising from dimers and other particle aggregates. Then, SERS measurements were performed using an excitation laser with a wavelength of 633 nm; it was found that even though the surface coverage diminished, the highest enhancements were obtained with the biggest nanoparticles, which was attributed to large size distributions that allowed to obtain various hot spots configurations and because the LSPR band was in resonance with the used excitation wavelength [91].

At this point, and to sum up the results obtained from some reports, Table 2 is presented to show the SERS properties of Ag/TiO_2_ NTs substrates. The table is composed of: Probe molecule; wavelength of the excitation source; limit of detection in molar concentration; recyclability, in the sense that the substrate was cleaned upon UV irradiation, how long it took to completely degrade the adsorbed molecules, and how many times was possible to use the substrate.

It is noteworthy to see that the silver nanoparticle–titanium dioxide nanotubes are able to degrade the adsorbed molecules upon UV irradiation, being an excellent choice for SERS because it allows multiple uses, eliminating the one-time use drawback of some substrates [94]. Moreover, Sun and co-workers report that photocatalytic degradation of the adsorbed molecules has no impact on the morphology of the substrate, as evidenced by XPS measurements. They found that the adsorbed molecules are degraded onto smaller ones (including carbon dioxide and water), which can be easily removed by washing the substrate [76]. Additionally, Chong and co-workers observed that once that the Ag/TiO_2_ NTs array is cleaned upon UV irradiation, it is possible to achieve reproducible signals on further SERS measurements, as performed on R6G molecules, demonstrating the reusability [94].

### 5.2. Gold Nanoparticles (Au NPs)

Gold nanoparticles (Au NPs) have been extensively studied as SERS substrates as they can be easily prepared either from chemical routes as the reduction of HAuCl_4_ with a proper reducing agent (sodium borohydride, trisodium citrate, ethylendiaminetetracetic acid, formaldehyde, hydrazine, hydroxylamine, polyols, oxalic acid, sugars, etc.), through biological methods that make use of plant extracts (*M. sativa*, *A. vera*, *C. gossypium*, among several others) or by physical methods such as laser ablation, vacuum sputtering or condensation methods [6,49,105,106,107]. They also present very good biocompatibility, low reactivity, and can be easily modified on their surfaces by chemical methods, aside from exhibiting a strong plasmonic response, which is strongly tied to changes in their surroundings and to the electronic structure of substrates where they are deposited [108]. Due to all those characteristics, Au NPs are of potential use in the development of biosensors, medical imaging, catalysis, among other applications [109]. Au NPs can be easily deposited on different types of materials such as glass slides, mesoporous silica, tin oxide thin films, silicon wafers, aluminum foil, copper, polymer films, paper, plastic, etc., using different methods such as centrifugation/sedimentation, ultrasonic dispersion, thermal annealing, wet immersion deposition, chemical spray pyrolysis, convective assembly through Coulombic interactions, sputtering, silane-immobilization, electrophoretic deposition, precision photoreduction, among several others [95,110,111,112,113,114].

#### Gold Nanoparticle Deposition on TiO_2_ NTs

Semiconductor-based SERS substrates are an alternative to metal-based SERS (Au, Ag, Cu), but have very low sensitivity. However, different types of porous TiO_2_ materials have been explored, as their cavities and complex 3D structure may be helpful to enhance SERS performance. Following that idea, a TiO_2_ nanofoam-nanotube array was prepared by tuning the anodic voltage and the electrochemical reaction time, via a two-step anodic oxidation process, and its SERS response for the detection of methylene blue showed an intense SERS effect due to cavity-enhanced Raman scattering [18]. Nanocomposites formed by incorporating noble metal nanoparticles into photocatalytic materials (plasmonic photocatalyst nanocomposites) have attracted the attention of several groups [61]. The formation of Au NPs composites with different types of semiconductor materials (ZnO, CeO_2_, TiO_2_, Fe_3_O_4_) has been explored as they can present improved sensing performance due to charge transfer from the metal oxide to the Au NPs, which can be used to modulate the electrochemical response, although it can also be used for designing SERS detectors. There are several reports that describe how the deposition of Au NPs on TiO_2_ NTs substrates enhances the surface electron interactions by plasmon resonance, improving the SERS response. Composites prepared by depositing TiO_2_ decorated with Au NPs on reduced graphene oxide nanosheets (TiO_2_-Au-rGO) have been also explored, which present high sensitivity for the detection of R6G up to 1.2 × 10^−10^ M [115]. It has been reported that even the specific crystalline phase of the TiO_2_ substrate (anatase, rutile or mixed crystal phase) can influence the intensity of the SERS response [80]. Using a 4-mercaptobenzoic acid as a molecular probe it was shown that mixed crystal structure favors a SERS enhancement with respect to pure anatase or rutile phases due to TiO_2_-to-molecule charge transfer mechanism and synergistic effect between rutile and anatase phases. The influence of crystalline phases was also explored by Zheng and co-workers who reported the enhancement of SERS sensitivity of TiO_2_ depending on the exposed facets [79]. They analyzed TiO_2_ single crystals with {0 0 1} and {1 0 1} facets co-exposed, and looked on the variations of the SERS response at different rations between the {0 0 1} and {1 0 1} facets; it was observed that molecules preferred to adsorb on defective {0 0 1} facets, although their SERS response was not proportional to that of enlarged {0 0 1} facets. These results may be useful to design better SERS responsive materials by controlling the crystallinity and facet heterogeneity.

Furthermore, as the TiO_2_ substrate keeps its photocatalytic properties, it can self-clean after UV-radiation exposition, allowing recyclability. Deposition of Au NPs on porous photoactive TiO_2_ nanostructures has been explored as a way to increase surface area and induce plasmonic coupling among adjacent Au NPs. Photocatalytic degradation can also be followed in situ in these types of systems by using Raman microspectroscopy as recently reviewed [2]. For example, a recyclable SERS substrate for multifold organic pollutants detection based on Au-coated TiO_2_ nanotube arrays was reported by Li and co-workers [116]. The TiO_2_ nanotubes were prepared by growing a ZnO nanorod array on a glass substrate, followed by coating ZnO nanorods with TiO_2_ (using (NH_4_)_2_TiF_6_ as a precursor) while, simultaneously, the ZnO nanorods are dissolved by the acids produced during the (NH_4_)_2_TiF_6_ hydrolysis. Finally, Au NPs were assembled on the TiO_2_ nanotube arrays by either the photo-deposition or the hydrothermal method. SERS spectra measurements on aqueous R6G solutions up to the 10^−6^ M concentration, as well as on solutions containing 4-chlorophenol (4-CP), persistent organic pollutants (POP), dichlorophenoxyacetic acid (2,4-D), and methyl-parathion (MP) in ethanol or methanol were performed. The nanocomposite arrays were highly sensitive, stable, reproducible, and recyclable, as after the organic pollutants are detected by the SERS effect, they can be easily degraded into clean inorganic molecules by UV irradiation of the substrates. In other examples of a recyclable SERS substrate, TiO_2_ films containing either nanotubes, nanolaces, or nanopores were coated with gold by vacuum thermal evaporation; the gold coating, which has a 20 nm thickness, was formed by spherical nanoparticles with diameters in the range of 20 nm homogeneously dispersed on the surface of the substrate [38]. The deposition process was performed at very slow rates (0.01 nm/s) to avoid the perturbation of the TiO_2_ nanostructure. R6G was used as a molecular probe to evaluate the SERS activity, showing a high response, which is directly relatable to the density of Au NP deposition on the substrate, as well as recyclability (the substrate self-cleans by exposition to UV irradiation). In a similar work, the group of Brognara and co-workers reported the preparation of a highly sensitive estradiol SERS sensor based on a porous TiO_2_ thin film coated with Au NPs [117]. The TiO_2_ thin film was deposited by pulsed laser deposition (PLD), which under different O_2_ pressure present controlled porosity; Au layers with thicknesses values of 3, 6, and 15 nm were then deposited by evaporation on top of the TiO_2_ films. After annealing at 500 °C, TiO_2_ recrystallized to the anatase phase and promoted the formation of the Au NPs, which presented different size distribution and the density depending on the porosity of the sample. The surface of the Au NPs was modified with a 17β-estradiol binding aptamer, in order to increase selectivity and affinity. The SERS sensor was tested against different aqueous solutions containing estradiol at concentrations from 1 nM to 1 mM, showing good sensitivity (from 1 nM) and a wide dynamic range (up to 100 µM). In other work, TiO_2_ nanosheets, decorated on both sides with Au NPs using a physical sputtering system to form a Au/TiO_2_/Au system, showed an improved SERS response by the induced plasmonic coupling resonance among Au NPs, presenting at the same time a large surface area for nanoparticles and molecules to interact. The SERS signal of the Au/TiO_2_/Au nanosheets was evaluated to detect 4-mercatptobenzoic acid, as a molecular probe: SERS spectra were obtained at different spots on the sensor, showing good stability, uniformity, and homogeneity of SERS signals. Later, adenine molecules were tested on label-free substrates, showing good sensitivity in the 10^−4^ to 10^−7^ M range [118].

Recently, Chen and co-workers [20] prepared a set of TiO_2_ nanotube arrays with well-defined morphologies by the electrochemical anodization method. Then, Au NPs were deposited using pulse current electrodeposition on the TiO_2_ NTs and their performance as SERS substrates for the detection of 4-mercatpobenzoic acid was measured. It was found that for Au NPs deposited on TiO_2_ NTs discontinuous surface arrays the SERS response was significantly higher than for those deposited on continuous TiO_2_ NTs arrays [20]. In other works, Zhang and co-workers prepared heterojunction materials by electrodeposition of Au NPs on the surface of WO_3_ nanoflowers growth on TiO_2_ nanotubes (Au/WO_3_/TiO_2_) which were evaluated as SERS detectors for dyes [8]. The fabrication of the nanocomposite started with the preparation of the TiO_2_ nanotube on a Ti foil by anodic oxidation, which was then used as substrates for the hydrothermal growth of WO_3_ nanoflowers by loading the WO_3_ seed solution on the TiO_2_ NTs sheet placed into a 50 mL Teflon-lined stainless-steel autoclave at 180 °C for 2 h, which was then annealed at 500 °C for 2 h after. Gold NPs were electrodeposited on the WO_3_/TiO_2_ substrates at different pulse sequences to control the density of deposited Au NPs. The influence of the WO_3_ layer, which presents a large surface area due to its flower-like 3D structure, enhanced the Raman response by a factor of 3.6 × 10^6^, with good stability and high uniformity in the SERS response. The nanocomposite was used for the detection of four dyes: R6G, crystal violet, alizarin, and malachite green, which were detected even at the nanomolar level [8].

Bimetallic systems, using Au NPs as core and an Ag shell, have been also explored due to the potential synergistic electromagnetic coupling between the two metals. The group of Wang et al. reported a recyclable microfluidic Au@Ag/TiO_2_ NTs SERS chip prepared by the in situ growth of Au@Ag NPs in the microchannels of the TiO_2_ NTs. First, a microfluidic chip containing a TiO_2_ NTs layer was carefully built. The channels were then filled with different concentrations of poly dimethyl diallyl ammonium chloride (PDDA), which was used to control the density of deposition of Au NPs. After deposition, a chemical silver-plating solution was poured through the microchannels, obtaining the desired microfluidic SERS chips with an integrated Au@Ag/TiO_2_ NTs layer [65]. Evaluation of the SERS response towards R6G achieved very good sensitivity (up to 10^−10^ M) and repeatability; charge transfer from TiO_2_, the Au@Au plasmon, and the adsorbed molecules contained in the 3D NTs structure enhanced the SERS response. The shape of the bimetallic nanoparticles affects the SERS response; in another work, core-shell Au@Ag nanocubes were prepared by the epitaxial method, showing a strong plasmonic coupling among the Au core and the Ag shell in the intra-junction space, which produces a stronger SERS enhancement in comparison to pure Ag nanocubes [119]. That work suggests that designing bimetallic nanoparticles with controlled morphologies, different shapes and complexities may be useful to design better systems for sensor or imaging applications. A summary of selected examples of SERS performance of Au NPs/TiO2 substrates is presented in Table 3.

### 5.3. Platinum Nanoparticles (Pt NPs)

Platinum nanoparticles are perhaps mostly known because of their catalytic behavior. Their large surface areas enable them to have outstanding performances that are exploited in the chemical industry for catalysis [120,121]. Although, since these nanostructures have free electrons, surface plasmon resonance effects can be observed as well, the activity in SERS has been demonstrated although rarely reported [16,121,122].

#### Platinum Nanoparticle Deposition on TiO_2_ NTs

Similar to silver nanoparticles, it is possible to modulate the properties of Pt NPs by controlling the synthesis conditions [120]. As an example, Cai and co-workers modified an anatase-phase TiO_2_ NTs substrate with polydopamine (PDA), whose functional groups served as anchors for platinum nanoparticles. Then, different PDA/TiO_2_ NTs substrates were submerged in solutions of H_2_PtCl_6_ of diverse concentrations, ranging from 0.1 × 10^−3^ to 0.8 × 10^−3^ M, under stirring and at 90 °C for 3 h. Upon SEM images it was found that, at the lowest concentration precursor, a small amount of nanoparticles were present on the surface of the nanotubes; by increasing the concentration to 0.4 × 10^−3^ M, it was possible to observe clusters of aggregated nanoparticles and at 0.8 × 10^−3^ M the nanoparticles aggregated and nearly blocked the nanotubes open ends. Thus, the density of the nanoparticles on the surface of the nanotubes increased with the precursor concentration. This had an impact on the SERS spectra of R6G, whose signals increased with the amount of nanoparticles until obtaining a maximum enhancement factor of 4.3 × 10^4^ at a precursor concentration of 0.4 × 10^−3^ M, attributed to the LSPR effect of the nanoparticles and the charge transfer of TiO_2_ [16].

Additionally, Dong and collaborators [123] studied the effect of varying the amount of Pt NPs on the SERS effect. First, they deposited MoS_2_ nanosheets onto TiO_2_ NTs to enhance the charge transfer. Then, Pt NPs were deposited using a cyclic voltammetry method in a solution of 0.5 M H_2_SO_4_, where the MoS_2_/TiO_2_ NTs sample served as working electrode, a Pt wire as a counter electrode, and Ag/AgCl as a reference electrode. The applied voltage ranged from −0.8 to 0 V and the scan rate was 0.2 V/s. This was performed with different numbers of cycles, from 500 to 2000. By SEM characterization it was observed that at 500 cycles no nanoparticles were deposited onto the substrate due to the low dissolution rate of platinum. With 1000 cycles, a small number of nanoparticles were deposited. At 1500 cycles, the nanoparticles accumulated on the sulfur-rich sites of the substrate, and with 2000 cycles the nanoparticles aggregated, which is undesirable for the photocatalytic activity because light cannot penetrate deeply into the substrate, and only a fraction of it is irradiated [123,124]. Finally, SERS measurements revealed that the best signals for R6G were obtained on the substrate prepared with 1500 cycles, which was in concordance with UV-vis spectra that showed the largest absorptions for this substrate. This allowed achieving an enhancement factor of 2.1 × 10^5^. The authors attributed the signal enhancement to the surface plasmon of the nanoparticles and to the vertical structure of the substrate which enabled fast electron transport [123].

Thus, it is demonstrated that other noble metals can be used for SERS applications but a disadvantage is that they do not produce as large enhancements as silver, gold, or copper. Actually, the enhancements factors obtained with other transition metals are estimated to be between 1 and 4 orders of magnitude [125]. Nevertheless, these arrays also show self-cleaning ability because Pt NPs are able to degrade the adsorbed species and trap the photogenerated electrons from TiO_2_ and impede the recombination [126].

In fact, Cai and co-workers were able to demonstrate the separation of photogenerated electron-hole pairs by measuring the current of the different substrates under light irradiation and at an applied potential of 0.26 V (vs. Ag/AgCl). Bare nanotubes had the lowest photocurrent, with a value of 0.003 mA/cm^2^ due to rapid electron-hole recombination. In contrast, when Pt NPs were present, the photocurrents increased greatly with values of 0.038, 0.046, 0.068, and 0.054 mA/cm^2^ for the substrates submerged in the H_2_PtCl_6_ solutions at concentrations 0.1 × 10^−3^, 0.2 × 10^−3^, 0.4 × 10^−3^, and 0.8 × 10^−3^ M, respectively, demonstrating that platinum nanoparticles are able to hinder the recombination of photogenerated species. This, in turn, served for the recyclability purpose because, upon UV irradiation, the signals from R6G nearly vanished after 150 min. In the same manner, Dong and co-workers [123] showed the self-cleaning ability of the Pt NPs/MoS_2_ NS/TiO_2_ NTs array, by irradiating the substrate for 90 min with UV light which resulted in the disappearance of the SERS signals of R6G. Therefore, the development of nanoplatforms of platinum nanoparticles and titanium dioxide nanotubes relies on the interest of building SERS substrates that can be reused [16].

## 6. Discussion

As we have reviewed, metal nanoparticles can be deposited on several supports by a variety of strategies: thermal evaporation, electron beam evaporation, ion sputtering, electrochemical, wet chemical, vapor-phase synthesis and photoreduction, hydrothermal deposition, etc. Certainly, at this point, there is a void in the research comparing preparation strategies for a given substrate or even in the reporting of parameters that could show advantages of these strategies such as EF, limit of detection, stability, accuracy, and precision, as we can see from Table 4 for R6G SERS analyses. For the deposition of metal nanoparticles on TiO_2_ NTs, some experimental approaches such as chemical and photoreduction are more accessible, in terms of reagents cost and equipment, and therefore more extensively used. Between these two, photochemical reduction is a more promising technique as it avoids impurities coming from reductant reagents. Another technique that involves higher cost in equipment but guarantees cleaner substrates is ion sputtering. This technique is also advantageous as it allows the controllable growth of nanoparticles. Electrochemical techniques such as pulsed current electrodeposition and cyclic voltammetry are also promising as they are able to control the density of deposited materials and impurities development is less likely to occur.

In terms of stability, at the desired nanometer size level required for the efficient performance of this technique, noble metal nanoparticles will undergo degradation and, from our point of view, there is not a significant difference in the reported stability for the metals (Ag, Au, Pt) considered in this review. In this sense, we observe a trend in semiconductors nanoengineering in order to enhance their SERS performance since they may present more stability and allow self-cleaning. To this respect, it is worth mentioning the review by Krajczewski and co-workers [5] where several semiconductors’ interesting features have been addressed.

With respect to the use of titania nanotubes as solid supports for SERS substrates, we have seen that their morphology possesses appropriate features for SERS such as a high surface area and a regular pattern surface that can hold nanoparticles with a higher number of hot spots. We envision that with nanoparticles of adequate size, the open ends of the nanotubes along with the gaps between the tubes can be tuned to improve metal nanoparticles arrangements to improve SERS analytical response. In addition, regarding nanotubes, another important aspect to be considered in their synthesis is their thermal annealing; since a mixture of anatase and rutile crystalline phases, with anatase in a higher ratio, usually around 85:15 (anatase:rutile), is found to improve charge transfer and therefore, SERS performance.

## 7. Conclusions

In this review, we have discussed the use of titania nanotubes as solid supports for plasmon metal nanoparticles to develop more effective SERS substrates. We have presented the different methodological approaches towards nanotubes, prepared by anodization, that meets the adequate features to disperse metal nanoparticles with an appropriate size and distribution to achieve a high SERS performance.

We have seen that both nanoparticles size and nanotubes morphology have to be tuned in order to find a proper nanoparticle distribution. For instance, small diameter nanoparticles may go inside the nanotubes and become isolated, but also very small diameter nanoparticles can be deposited on the top of the nanotubes forming rings with deposited nanoparticles with high EF. Another interesting observation is the adequate performance that arose from nanoparticle deposition within the gaps between the nanotubes that had this morphology.

As far as deposition is concerned, several chemical and physical methods have been performed to cover titania nanotubes with metal nanoparticles where time and number of deposition cycles have played, as expected, a key influence on the growth and dispersion patterns on the nanotubes. These variables must be optimized for the particular deposition technique, which is an area of opportunity for more systematic research to be conducted in the field.

Finally, as the self-cleaning ability of this nanostructured photocatalytic metal oxide is driving the research on its SERS applications, the efficiency of this process still needs to be evaluated under the balance of cost-benefit and, certainly, deeper investigation on the photocatalytic conditions for this particular application is still on the road.

## Figures and Tables

**Table 1:** Nanostructured semiconductors for SERS applications.

Semiconductor	Synthesis Method	Probe Molecule	Enhancement Factor	LOD	Reference
CdTe quantum dots		4-Mpy	10^4^		[50]
Cu_2_O nanospheres		4-MBA	≈10^5^		[51]
MoS_2_ monolayer	APCVD	4-Mpy	3.8 × 10^5^		[52]
SiO_2_ particles		CV	2.2 × 10^4^		[53]
SnO_2_ octahedral nanoparticles	Self-assembly	4-MBA	10^3^		[54]
TiO_2_ inverse opal photonic microarray	Casting and calcination	MB	2 × 10^4^	6 × 10^−6^ M	[55]
TiO_2_ nanosheets		4-MBA	1.86 × 10^6^		[56]
TiO_2_ nanoparticles	Sol-hydrothermal	4-MBA	3.5 × 10^3^		[57]
ZnO nanosheets	Self-assembly	4-MBA	10^3^	1 × 10^−6^ M	[58]
ZnSe nanoparticles	MBE	4-Mpy	2 × 10^6^		[59]
ZnS nanocrystals		4-Mpy	10^3^		[60]

LOD—limit of detection; 4-MPy—4-lercaptopyridine; 4-MBA—4-mercaptobenzoic acid; RISA—recrystallization-induced self-assembly; APCVD—ambient pressure chemical vapor deposition; CV—crystal violet; MB—methylene blue; MBE—molecular beam epitaxy.

**Table 2:** SERS properties of Ag NPs/TiO_2_ NTs substrates.

Probe Molecule	Excitation Wavelength	LOD (M)	EF	Recyclability	Reference
Formaldehyde	532 nm	1.21 × 10^−7^		3 h, 3 times	[90]
2-mercaptobenzoxazole	514 nm	~10^−9^	2.26 × 10^8^	20 min, 3 times	[89]
Rhodamine G	633 nm	10^−8^		140 min, 3 times	[94]
Rhodamine G	532 nm	10^−7^		20 min, 3 times	[104]

FA—formaldehyde; MBO—2-mercaptobenzoxazole; R6G—rhodamine G.

**Table 3:** Selected examples of SERS performance of Au NPs/TiO_2_ substrates as sensors towards some selected molecules.

Molecular Probe	Excitation Wavelength	LOD (M)	EF	Recyclability	Reference
4-CP	785 nm	1 × 10^−9^	---------	30 min, three times	[116]
R6G	514 nm	1 × 10^−5^	5 × 10^4^	270 min, 4 times	[38]
4-MBA	647 nm	1 × 10^−9^	1 × 10^7^	----------------	[118]
Estradiol	633 nm	1 × 10^−9^	1 × 10^6^	----------------	[117]

R6G—rhodamine 6G; 4-MBA—4-mercaptobenzoic acid; 4-CP—4-chlorophenol; LOD—limit of detection; EF—enhancement factor.

**Table 4:** Rhodamine 6G SERS analyses on different substrates.

Substrate	Synthesis Method	EF	LOD	Relative Standard Deviation	Stability	Reference
Ag NPs-coated CP	Chemical Reduction on CP by hydrazine		10^−11^ M	7.6% six for 6 batches	No obvious change after 1 month	[127]
Ag NPs—Cu grid	Chemical reduction, drop casting deposition, glow discharge treatment on Cu-grids	6.1 × 10^5^	240 ppb		5–10% signal reduction after 3 weeks	[128]
Ag NPs/TiO_2_ NTs	Photochemical reduction		10^−8^			[94]
Ag NPs/TiO_2_ NTs	Chemical reduction by Sn^2+^		10^−7^			[104]
Au NPs film	EBE Au deposition on Si wafer followed by ER	2.45 × 10^8^	7.08 × 10^−11^ M	6.88% for 12 measurements	26.5% signal reduction in 30 days	[129]
Au NPs/TiO_2_ nanopores	Au evaporation on anodized Ti	5 × 10^4^	1 × 10^−5^ M			[38]
Pt nanoaggregates on Si wafers	Pt solution dropping on Si wafer	5 × 10^4^				[130]
Pt@TiO_2_ NTs	Reduction at 90 °C of adsorbed Pt ions on PDA-modified TiO_2_ NTAs	4.3 × 10^4^	~10^−8^ M			[16]
Pt NPs/MoS_2_/TiO_2_NTs	Pt NPs deposited by CV on MoS_2_ nanosheets deposited on TiO_2_NTs	2.5 × 10^5^				[123]
Fe_2_O_3_ NPs/N-rGO	Fe_2_O_3_ NPs grown in situ on N-rGO		5 × 10^−7^ M	<9.43% for 10 measurements		[131]
Partially oxidized MoS_2_ nanosheets	Thermal oxygen incorporation in MoS_2_	1.4 × 10^5^	10^−7^ M			[132]
Cu_2_O mesoporous spheres	Recrystallization induced self-assembly	~10^5^	10^−9^ M			[133]
TiO_2−x_ nanorod films	Hydrothermal method	1.8 × 10^4^	10^−6^ M		Reduced signals after 2 months	[134]

CP—cellophane; N-rGO—nitrogen-doped reduced graphene oxide; EBE—electron beam evaporation; ER—electrochemical roughening; PDA—polidopamine; CV—cyclic voltammetry.

## Data Availability

Not applicable.
